# Eye gaze differences in school scenes between preschool children and adolescents with high-functioning autism spectrum disorder and those with typical development

**DOI:** 10.1186/s13030-020-00203-w

**Published:** 2021-01-18

**Authors:** Yuko Ishizaki, Takahiro Higuchi, Yoshitoki Yanagimoto, Hodaka Kobayashi, Atsushi Noritake, Kae Nakamura, Kazunari Kaneko

**Affiliations:** 1 Department of Pediatrics, Kansai Medical University, Hirakata, Osaka Japan; 2 Department of Physiology, Kansai Medical University, Hirakata, Osaka Japan; 3 Division of Behavioral Development, Department of System Neuroscience, National Institute for Physiological Sciences, National Institutes of Natural Sciences, Okazaki, Japan

**Keywords:** Eye gaze behavior, High-functioning autism spectrum disorder, Classroom, Typical development, Preschool children, Adolescents

## Abstract

**Background:**

Children with autism spectrum disorder (ASD) may experience difficulty adapting to daily life in a preschool or school settings and are likely to develop psychosomatic symptoms. For a better understanding of the difficulties experienced daily by preschool children and adolescents with ASD, this study investigated differences in eye gaze behavior in the classroom environment between children with ASD and those with typical development (TD).

**Methods:**

The study evaluated 30 children with ASD and 49 children with TD. Participants were presented with images of a human face and a classroom scene. While they gazed at specific regions of visual stimuli, eye tracking with an iView X system was used to evaluate and compare the duration of gaze time between the two groups.

**Results:**

Compared with preschool children with TD, preschool children with ASD spent less time gazing at the eyes of the human face and the object at which the teacher pointed in the classroom image. Preschool children with TD who had no classroom experience tended to look at the object the teacher pointed at in the classroom image.

**Conclusion:**

Children with ASD did not look at the human eyes in the facial image or the object pointed at in the classroom image, which may indicate their inability to analyze situations, understand instruction in a classroom, or act appropriately in a group. This suggests that this gaze behavior of children with ASD causes social maladaptation and psychosomatic symptoms. A therapeutic approach that focuses on joint attention is desirable for improving the ability of children with ASD to adapt to their social environment.

## Background

Autism spectrum disorder (ASD) is a neurodevelopmental disorder characterized by impairments in social interaction and communication, as well as repetitive and restricted behaviors, although intellectual development is not always delayed [1]. Psychosomatic symptoms are prevalent among children with ASD because they often experience social maladaptation, especially school refusal [2]. Children with ASD have restricted interests and behaviors that may cause them to struggle with behaving like other children who have typical development (TD). Their behaviors are often misunderstood, and they may be scolded by their teachers or bullied by their friends [2]. For children with ASD, school refusal occurs at a younger age than for those with TD [3]. Children with ASD are also prone to hyperacusis such that the sounds of the school environment may be a scary experience for them, resulting in an increased prevalence of social maladaptation and school refusal compared with children with TD [2]. In addition, because children with ASD have difficulty expressing their feelings, they often develop psychosomatic symptoms [2].

To prevent the development of psychosocial symptoms, it is essential to detect symptoms of ASD at an early stage and to collaborate with school teachers. Increasing evidence suggests that, in contrast to individuals with TD, those with ASD exhibit characteristic eye gaze behavior [4–11], including more attention to a person’s mouth than eyes [5, 6]; less attention to children playing [9] and to the social activities of others, with more attention on background objects instead [11]; and downward-looking fields of view [6].

In addition to faces and classroom scenes are among the most important social and visual images used to assess the attention of children because they spend most of their time in classrooms. The classroom scene is also characterized by situations in which a teacher points to an object, such as a whiteboard or another display, in which “joint attention” [12, 13] is required simultaneously to both stimuli (the teacher and the object) rather than direct communication between the teacher and child. Thus, the first hypothesis of this study was that the characterization of eye gaze behavior of children with ASD using classroom scenes can demonstrate the reasons for their difficult experiences at preschool or school [14]. The first analysis in this study focused on eye gaze behavior in a school classroom scene as well as on a widely studied human face. However, it was unclear whether the children’s eye gaze behavior in the classroom was caused by an inability for joint attention or by the effect of learning.

The second hypothesis of this study was that the eye gaze pattern in a classroom setting may emerge as a result of the subjects’ nature of scene analysis rather than from the experience of attending the class. If so, then preschool children with ASD and those with TD who never had classroom experiences should respond differently. Thus, this study compared the eye gaze behavior of children with ASD to that of children with TD in discontinuous age groups of preschool children (age 3–6 years) and adolescents (age 11–15 years), but not elementary school children (age 7–10 years in grades 1–4). This design was based on the thought that younger elementary school children are learning where to look in the classroom and during this stage it is not possible to judge whether their gaze behavior was due to an inability of joint attention or the effect of learning. Therefore, younger elementary school children were excluded and the study focused on comparing preschool children who have not experienced the classroom setting with adolescents who have more than 5 years of classroom experience.

## Methods

### Participants

This study evaluated 79 Japanese students: 30 high-functioning children with ASD and 49 children with TD. Participants from two discontinuous age groups were included: preschool children age 3–6 years with no previous classroom experience and adolescents age 11–15 years who had attended elementary school or junior high school. The study compared the eye gaze behavior of four groups: 25 preschool children with TD (7 boys), 12 preschool children with ASD (9 boys), 24 adolescents with TD (11 boys), and 18 adolescents with ASD (11 boys). Exclusion criteria were any past or present psychiatric illness; difficulties in eye movement or visual function; and inability to accomplish the 10-min experiment described in the Methods section. Written informed consent for all student–participants was obtained from their parents.

### Criteria for ASD and TD

High-functioning ASD was diagnosed by specialists in the field of pediatric neurology and/or developmental pediatrics according to the following criteria:

- Autistic disorder or pervasive developmental disorder based on the *Diagnostic and Statistical Manual of Mental Disorders*, fourth edition, text revision [15]
- A full scale intelligence quotient (FSIQ) score of ≥70 in the *Wechsler Intelligence Scale for Children*, fourth edition [16], for children age > 5 years and a developmental quotient (DQ) of ≥70 in the Kyoto Scale of Psychological Development [17] for children age < 5 years
- A score of ≥25.5 in the Childhood Autism Rating Scale (CARS) [18] or a score above the cutoff value for the relevant age group in the *Parent-Interview ASD Rating Scale*, text revision (PARS-TR) [19].

The CARS score indicates the severity of autism and originally considered that scores < 30 indicated no autism and scores > 30 indicated mild-to-moderate or severe autism [18]. However, Tachimori et al. [20] recently reported that children diagnosed with Asperger syndrome could be distinguished from those without ASD using cutoff values of 25.5 versus 26.0, respectively. The present study also used this criterion to differentiate children with ASD from children with TD (CARS score 25.5 vs. 26.0, respectively). The Japanese version of the PARS-TR [19] was administered in a semi-structured interview with a parent or family member of the child. This scale evaluates both the current symptoms and the most pronounced symptoms during infancy, defined by the PARS-TR peak symptoms scale. A significant correlation exists between PARS-TR scores and Autism Diagnostic Interview-Revised (ADI-R) scores, particularly between qualitative abnormalities in the reciprocal social interaction component in the ADI-R score and the social communication component in the PARS-TR score [21].

### Ethical approval

The research was approved by the Ethics Committee of Kansai Medical University (No. 1100).

### Experimental design

**Fig. 1:**
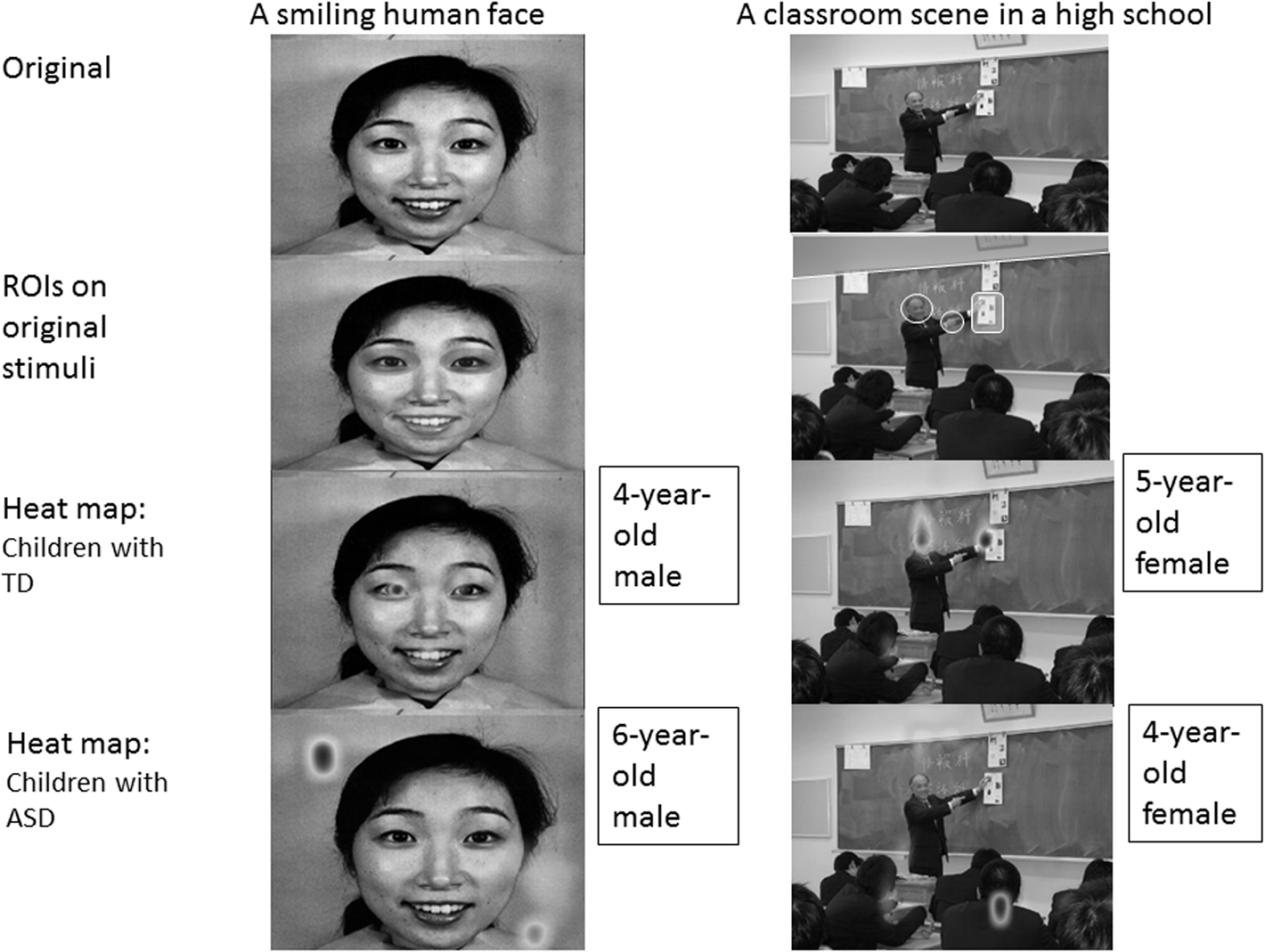
Visual stimuli and ROIs. Row 1, original stimuli; row 2, original stimuli with visual areas as ROIs; rows 3 and 4, representative gaze patterns of children with TD (row 3) and ASD (row 4) shown as heat maps

Experiments were conducted in a quiet, well-lit room at Kansai Medical University Medical Center. Participants were seated in front of a 48 × 30 cm monitor for the presentation of visual stimuli, and their chins were placed on a chin rest to minimize head movement. The distance between the monitor and the chin rest was 60 cm. Partitions were placed to ensure that only the monitor was within the participant’s field of vision. On the monitor, two social images—a smiling human face and a classroom scene in a high school setting (Fig. 1)—were presented sequentially, once for each image, with no sound. Each stimulus was shown for 9 s followed by an intertrial interval of 1 s. The duration of the whole experiment was approximately 10 min (Fig. 1, row 1). The participants were instructed to freely watch the static visual images on the monitor. The eye gaze position was measured at 250 Hz using an infrared camera attached to the bottom of the monitor (iView X RED, SensoMotoric Instruments, Teltow, Germany). Eye tracking data were analyzed using a customized software program written in MATLAB (MathWorks, Natick, MA, USA).

Of the 79 participants, five could not complete the 10-min experiment (4 for a human face; 1 participant for a classroom scene) and thus were excluded from the following analyses.

### Statistical analysis

To compare the eye gaze behavior of children with TD and children with ASD and that of preschool children and adolescents, the testing first identified the visual areas that were regions of interest (ROIs) (Fig. 1, row 2). The ROIs were set as the eyes and mouth for the human face image and the face, pointing finger, and object pointed at by the teacher in the classroom image. The eye gaze time of preschool children with ASD and those with TD was compared for each ROI using the Welch t-test. The same was done for adolescents with ASD and those with TD. Within the 9 s of each stimulus presentation, the duration of the gaze in each ROI was measured. Statistical analyses were performed using the statistical software SPSS version 22.0 (IBM Corp., Armonk, NY, USA).

## Results

### Patient characteristics

For the developmental assessment of patients with ASD, the means ± standard deviations were as follows: preschool DQ 87.3 ± 14.5 and adolescent FSIQ 96.0 ± 13.7. The mean CARS score of participants with ASD was 28.1 ± 3.6. The mean PARS-TR peak symptom scale score was 30.4 ± 5.4, and the mean current symptom score was 21.0 ± 9.8. These scores were higher than both the cutoff value of 25.5 for CARS for 9 preschool and 13 for elementary school students and the cutoff value for the relevant age group (adolescent/adult) for PARS in 20 junior high school students. These findings mean that autism symptoms were obvious in patients with ASD.

### Characteristics of eye gaze behavior

Figure 1 illustrates the representative eye gaze patterns of children with TD and ASD. For the human face stimulus (Fig. 1, left column), TD children held their gaze longer on the eyes and mouth (row 3), whereas ASD children gazed longer on the points between eyes and mouth or the wall, and two participants with ASD never looked at the face (row 4). For the classroom scene (right column), TD children gazed longer at the areas near the teacher’s face and at the object to which the teacher pointed. Conversely, ASD children tended to gaze longer at a location irrelevant to the class, such as the center of the screen, the pencil case on the desk, or the wall. These ASD children almost never looked at the teacher’s face or the object that was pointed out.

**Fig. 2:**
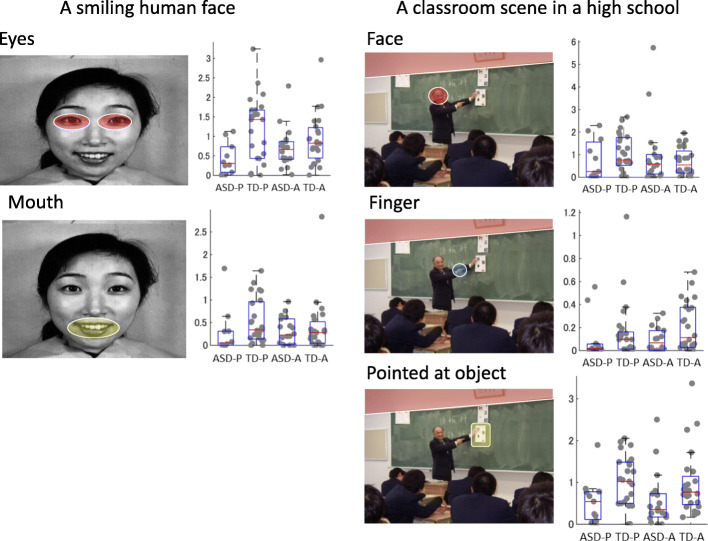
Visual stimuli and box-and-whisker plot of the ROIs for gaze duration on the human face and classroom scenes between children with ASD and those with TD. ASD-Y, preschool children with ASD (*n* = 10); TD-Y, preschool children with TD (*n* = 24); ASD-O, adolescents with ASD (*n* = 18); TD-O, adolescents with ASD (*n* = 23)

Figure 2 presents a box and whisker plot of eye gaze duration for the ROIs of the human face and the classroom scene. A statistically significant neurodevelopmental effect on the duration of the gaze was observed (Table 1). The Welch *t* test revealed a significant difference between TD and ASD preschool children when they gazed at the object pointed at [t(21) = 4.83, *p* = 0.039], whereas adolescents with TD tended to look at the object pointed at longer than did those with ASD [t(39) = 3.20, *p* = 0.081].

**Table 1:** Comparison of gaze duration on facial features and objects between children with ASD and those with TD

	A smiling human face			A classroom scene in a high school		
	Eyes	Mouth		Face	Finger	Pointed-at object
Preschool Children						
TD (*n* = 24)	1.18 ± 0.14	0.53 ± 0.10	TD (*n* = 25)	1.06 ± 0.19	0.16 ± 0.05	1.00 ± 0.13
ASD (*n* = 10)	0.44 ± 0.21	0.31 ± 0.16	ASD (*n* = 11)	0.78 ± 0.29	0.11 ± 0.06	0.55 ± 0.20
Adolescents						
TD (*n* = 23)	0.92 ± 0.14	0.39 ± 0.10	TD (*n* = 24)	0.70 ± 0.20	0.21 ± 0.04	0.97 ± 0.14
ASD (*n* = 18)	0.71 ± 0.16	0.31 ± 0.12	ASD (*n* = 18)	1.01 ± 0.23	0.10 ± 0.05	0.58 ± 0.16
Welch’s t-test						
Preschool	t (31) = 11.7	t (16) = 1.31		t (18) = .83	t (25) = .45	t (21) = 4.83
ASD vs TD	*p* = .002	*p* = .269		*p* = .374	*p* = .507	*p* = .039
Adolescents	t (39) = 1.42	t (34) = .30		t (21) = .73	t (35) = 5.21	t (39) = 3.20
ASD vs TD	*p* = .241	*p* = .589		*p* =. 402	*p* =. 029	*p* =. 081

Data are presented as mean ± standard deviation of the number of seconds

*TD* Denotes typical development

*ASD* Denotes autistic spectrum disorder

Notably, despite the lack of classroom experience for preschool children with TD, they looked at the teacher’s face and the pointed-at object. Moreover, although the shortened gaze duration in preschool children with ASD was evident for the smiling human face image, the difference was not significant for the same face presented within the classroom image. Preschool children with TD gazed significantly longer at the eyes of the human face image than did preschool ASD children [t(31) = 11.7, *p* = 0.002]. No significant difference was observed between TD and ASD adolescents for gaze duration at the human face image [t(39) = 1.42, *p* = 0.241].

## Discussion

Preschool children with TD looked at the eyes of the human face and the object pointed at in the classroom scene longer than did adolescents with ASD. The most beneficial result of this study was that preschool children with TD—even those with no previous classroom experience—looked at the object to which the teacher pointed in the classroom scene, which indicated that they may understand others’ intentions. This finding is in strong contrast to the eye gaze behavior of adolescents with ASD who had experience attending school. These results suggest that preschool children with TD and no classroom experience are equipped with the ability for joint attention in the classroom setting; however, this ability has not been acquired by adolescents with ASD. The finding that children with ASD who do not look at the object pointed at by the teacher is helpful and may lead to early detection of ASD in young children before preschool age, which would be beneficial for developing intervention strategies for such children.

Joint attention is a social communicative skill developed in early childhood in which two individuals use gestures and gaze to share attention with respect to interesting objects or events. This skill plays a vital role in social and language development [13, 22]. Children with ASD spent less time gazing at the eyes of the human face and at the object pointed to by the teacher and spent more time gazing at irrelevant areas, such as a pencil case on the desk in the school classroom scene. This behavior is consistent with a previous report by Noris et al. [6] in which children with ASD did not understand others’ intentions if the intentions were implied by eye gaze rather than by language, evident in the fact that ASD children did not look toward objects that were pointed at. Therefore, it is likely that children with ASD have difficulty in understanding interpersonal communication and acting appropriately in specific situations when in groups, resulting in social maladjustment such as school refusal.

The authors of this study propose that these characteristics also apply in classroom environments, resulting in difficulty in school performance for students with ASD. If lower school performance of children with ASD, despite a high intelligence quotient, is linked to impairment of joint attention in the classroom, then an educational program focused on joint attention would improve the school life of children with ASD. In the field of education, the results of this study may be useful when applying for funding to support lecture and classroom modifications for children with ASD. Even adolescents with ASD spent less time looking at the object that was pointed at, which suggests that instructing them to look at the teacher’s face or at what the teacher is pointing at so that they can understand what others require and how they are expected to act would be beneficial and might improve their social adjustment.

This study is limited in that it only describes the difference between children with TD and those with ASD in the duration of gaze on human facial features or specific objects. To determine how well students understand pointing behavior, the authors plan to analyze joint attention abilities in a future study.

## Conclusion

The differences in eye gaze behavior of preschool children with ASD and those with TD suggest that eye gaze behavior analysis could be used as an objective assessment for the early diagnosis of ASD in preschool children. Thus, this study highlights the future applicability of using eye gaze behavior as both a screening tool and in an educational and therapeutic approach for children with ASD. To treat their psychosomatic symptoms, it is important to know the features of eye gaze behavior of children with ASD in the classroom. An educational and therapeutic approach that focuses on joint attention in the classroom is desirable to improve the social adaptation of children with ASD.

## Data Availability

All data generated or analysed during this study are included in this published article and its supplementary information files.

## Abbreviations

ADI-R: Autism Diagnostic Interview-Revised
ASD: Autism spectrum disorder
CARS: Childhood Autism Rating Scale
DQ: Developmental quotient
FSIQ: Full scale intelligence quotient
PARS-TR: Parent-Interview ASD Rating Scale, text revision
ROI: Region of interest
TD: Typical development
